# P-643. Nationwide trends in respiratory syncytial virus (RSV) vaccinations and RSV vaccination efficacy in adults ages 60 and older

**DOI:** 10.1093/ofid/ofaf695.856

**Published:** 2026-01-11

**Authors:** Kathryn Lang, David Alfego, Min Kyung Lee, Laura Gillim, Charles M Walworth, Suzanne Dale, Colm Smart, Ruth Carrico, Payman Ghasemi

**Affiliations:** VaxCare, San Marcos, CA; Labcorp, Burlington, North Carolina; Labcorp, Burlington, North Carolina; Labcorp, Burlington, North Carolina; Monogram Biosciences/LabCorp, Laguna Beach, CA; Labcorp, Burlington, North Carolina; VaxCare LLC, Hooksett, New Hampshire; Norton Healthcare, Louisville, Kentucky; VaxCare, San Marcos, CA

## Abstract

**Background:**

Respiratory syncytial virus (RSV) vaccines were approved in 2023 and recommended for adults over the age of 75 and adults aged 60 – 74 at high risk of severe RSV infection. In this study, we investigated nationwide RSV vaccine administration and the efficacy of RSV vaccines in adults aged over 60.

**Figure 1.**
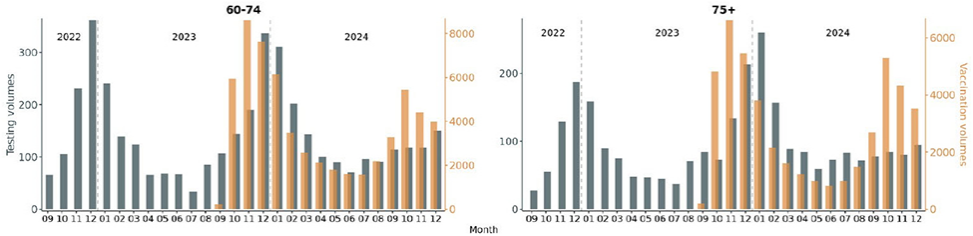
Monthly RSV vaccinations and testing volumes in adults 60 - 74 years of age and in adults 75+ from September 2022 to December 2024. Gray bars and the left y-axis indicate RSV testing volumes. Orange bars and right y-axis indicate RSV vaccination volumes.

**Figure 2.**
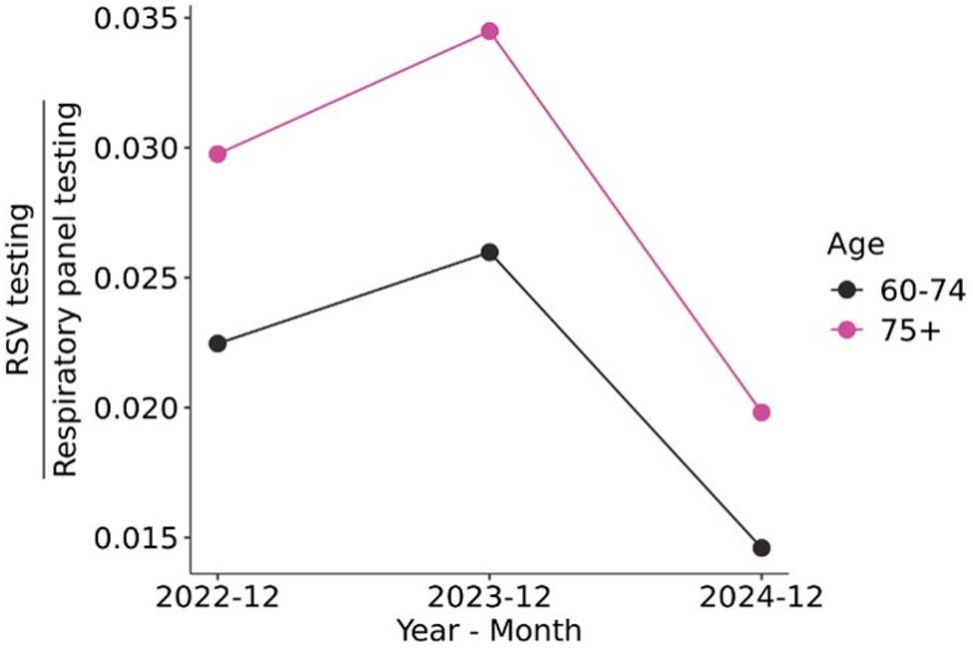
RSV testing volumes normalized to all respiratory panel testing volumes in December of 2022 – 2024 in adults 60 – 74 years of age and in adults 75+

**Methods:**

We conducted an observational analysis of two nationwide, deidentified crossover datasets of 107,035 RSV vaccination events from September 2023 to December 2024 and 6,660 RSV diagnostic testing from September 2022 to December 2024 in adults over the age of 60 who were administered seasonal vaccinations. Time-to-event analysis was conducted for 110 adults over age 60 with RSV diagnostic testing performed at Labcorp within one year of vaccination.

**Figure 3.**
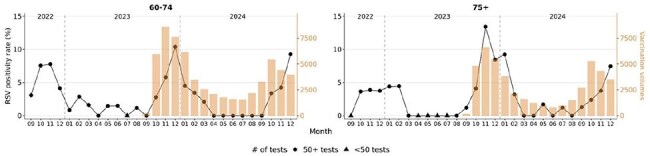
Monthly RSV positivity rates and vaccination volumes in adults 60 – 74 years of age and in adults 75+ from September 2022 to December 2024. Line plot indicates RSV positivity rates. Orange bars and right y-axis indicate RSV vaccination volumes. Shapes indicate the number of diagnostic tests performed per month.

**Figure 4.**
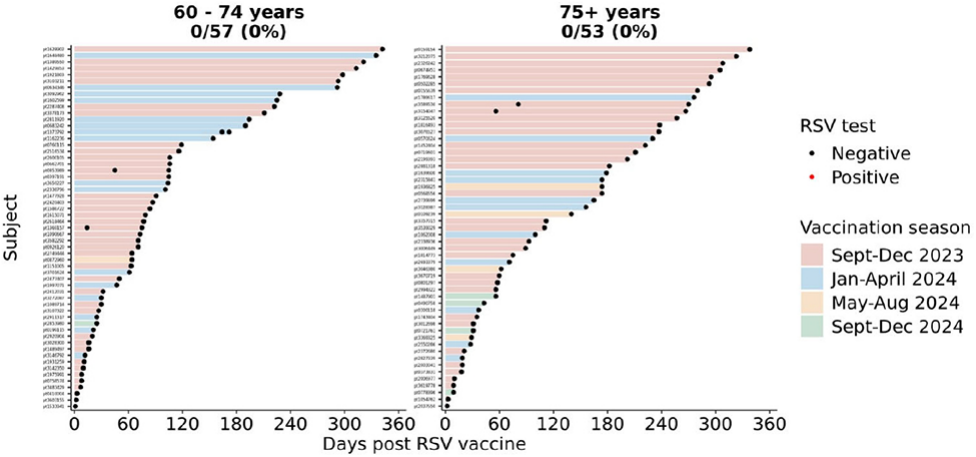
RSV diagnostic testing results within one year of RSV vaccination in 57 adults o60 – 74 years of age and in 53 adults 75+. Bars are colored by when each patient received the RSV vaccine.

**Results:**

Peak uptake of RSV vaccines occurred in November 2023 in the 2023 – 2024 RSV vaccination season and in October 2024 in the 2024 – 2025 RSV vaccination season in adults aged 60 – 74. For adults 75+, peak vaccine uptake occurred in November 2023 and October 2024 (Fig 1). Peak RSV testing in adults aged 60 – 74 occurred in December. Peak RSV testing in adults 75+ occurred in December 2022 and in January and December 2024. Peak uptake of RSV vaccines occurred 1 – 2 months prior to the peak RSV testing months. Testing volumes in peak testing month of December decreased by 43.8% in adults aged 60 – 74 and by 42.5% in adults 75+ 2024 from 2023 (Fig 2). Peak RSV positivity rates in 2023 – 2024 RSV vaccination season occurred in December in adults aged 60 – 74 (9.3%) and adults aged 75+ (7.4%; Fig 3). Vaccine uptake peaked 1 – 2 months prior to peak RSV positive months. There were no breakthrough RSV infections in 57 adults aged 60 – 74 or in 53 adults aged 75+ with RSV diagnostic testing within one year of vaccination (Fig 4).

**Conclusion:**

Our data demonstrate a potential correlation between the introduction of RSV vaccines and a reduction in RSV diagnostic testing in older adults. Furthermore, our data suggests that RSV vaccination is highly protective for RSV infection with zero breakthrough infections in a limited dataset. Further longitudinal study is warranted.

**Disclosures:**

Kathryn Lang, MD, PhD, VaxCare: Advisor/Consultant David Alfego, PhD, Labcorp: Employee|Labcorp: Employee|Labcorp: Stocks/Bonds (Public Company) Min Kyung Lee, PhD, Labcorp: Employee|Labcorp: Stocks/Bonds (Public Company) Laura Gillim, PhD, Labcorp: Stocks/Bonds (Public Company) Charles M. Walworth, MD, Labcorp: Employee|Labcorp: Stocks/Bonds (Public Company)|Labcorp: Stocks/Bonds (Public Company) Suzanne Dale, PhD, Labcorp: Stocks/Bonds (Public Company) Colm Smart, MBA, VaXcare: Stocks/Bonds (Private Company) Ruth Carrico, PhD, DNP, APRN, VaxCare: Advisor/Consultant Payman Ghasemi, PhD, VaxCare: Grant/Research Support

